# ‘Whip from the hip’: thigh angular motion, ground contact mechanics, and running speed

**DOI:** 10.1242/bio.053546

**Published:** 2020-10-21

**Authors:** Kenneth P. Clark, Christopher R. Meng, David J. Stearne

**Affiliations:** 1 Department of Kinesiology, West Chester University of Pennsylvania, West Chester, PA 19383, USA; 2 Department of Chemical and Biomolecular Engineering, University of Notre Dame, Notre Dame, IN 46556, USA

**Keywords:** Bipedal gait, Locomotor control, Sprinting

## Abstract

During high-speed running, lower limb vertical velocity at touchdown has been cited as a critical factor needed to generate large vertical forces. Additionally, greater leg angular velocity has also been correlated with increased running speeds. However, the association between these factors has not been comprehensively investigated across faster running speeds. Therefore, this investigation aimed to evaluate the relationship between running speed, thigh angular motion and vertical force determinants. It was hypothesized that thigh angular velocity would demonstrate a positive linear relationship with both running speed and lower limb vertical velocity at touchdown. A total of 40 subjects (20 males, 20 females) from various athletic backgrounds volunteered and completed 40 m running trials across a range of sub-maximal and maximal running speeds during one test session. Linear and angular kinematic data were collected from 31–39 m. The results supported the hypotheses, as across all subjects and trials (range of speeds: 3.1–10.0 m s^−1^), measures of thigh angular velocity demonstrated a strong positive linear correlation to speed (all *R*^2^>0.70, *P*<0.0001) and lower limb vertical velocity at touchdown (all *R*^2^=0.75, *P*<0.001). These findings suggest thigh angular velocity is strongly related to running speed and lower limb impact kinematics associated with vertical force application.

## Abstract (precis)

**Summary:** This investigation examined running speed, thigh motion and vertical force determinants. Results suggest thigh angular velocity is strongly correlated to speed and the lower limb impact kinematics underlying force application.

## INTRODUCTION

Whether at sub-maximal or maximal intensity, maintaining a constant forward running speed presents several mechanical challenges. Among these requirements, steady-speed running necessitates that the net three-dimensional acceleration of the center of mass (COM) over an entire stride cycle is zero. Therefore, the horizontal and lateral forces must integrate to zero during each stride, and the average vertical ground reaction force over a complete stride cycle must equal the body's weight (Raibert, 1986). Although a wealth of experimental research has investigated human locomotor performance (Blickhan, 1989; Farley et al., 1993; McMahon and Cheng, 1990; Nagahara et al., 2014; Rabita et al., 2015; Seyfarth et al., 2003; Weyand et al., 2000), a complete description of the mechanics that runners select to satisfy the demands of high-speed running has not yet been completely established. Therefore, further experimental investigation is warranted, to explore several important aspects of running mechanics.

Nearly a half-century of research has examined vertical force application during running (Cavagna, 1975; Cavanagh and Lafortune, 1980; Clark et al., 2017; Kuitunen et al., 2002; Munro et al., 1987; Nilsson and Thorstensson, 1989; Weyand et al., 2000, 2010). As speed increases and ground contact time decreases, the average vertical force applied during ground contact must increase to achieve the vertical impulse (force×time) necessary to support the body and rebound the COM into the next step. Recent research has demonstrated that faster speeds are typified by larger vertical forces primarily during the first half of ground contact (Bezodis et al., 2008; Clark and Weyand, 2014; Kuitunen et al., 2002), as vertical force application during the second half of ground contact is similar between sprinters and non-sprinters (Clark and Weyand, 2014). The large forces in the first half of ground contact can be explained by a Newtonian force–motion relationship (Bobbert et al., 1991), linking the sharp rising edge of the vertical force waveform observed at faster speeds to the rapid impact deceleration of the lower limb upon touchdown (Clark et al., 2017; Nigg et al., 1987).

In addition to lower-limb impact mechanics, proximal kinematic inspection (thigh segments) may provide additional insight. Previous publications on bipedal locomotion have suggested that limbs function as harmonic oscillators with torsion springs at the hip and scissor-like thigh angular motion (McGeer, 1990). In this investigation, we present a framework of thigh angular motion during the gait cycle (see Materials and Methods). Reciprocal oscillations at the hip result in angular movement patterns between the limbs, with one thigh flexing while the other extends. These angular motions are critical for facilitating steady forward speed (Raibert, 1986). Harmonic angular limb motion has been observed not only in human runners (Novacheck, 1998; Sides, 2015), but also in avian bipedal locomotion (Blum et al., 2014; Rubenson et al., 2007) and models of running robots (McGeer, 1990; Thompson and Raibert, 1989).

Furthermore, measurements of leg angular velocity have been positively related to running speed in human sprinting (Belli et al., 2002; Kivi et al., 2002; Kunz and Kaufman, 1981; Mann and Herman, 1985; Mann and Murphy, 2018; Miyashiro et al., 2019) and cited as a critical factor for running speed in robotic legged locomotion (Thompson and Raibert, 1989). However, despite prior evidence linking faster running speeds to both greater vertical ground reaction force and leg angular velocity, the connection between these two factors has not yet been fully explored across a range of speeds and runners. Theoretically, increased thigh angular velocity is not only necessary to satisfy the kinematic demands of faster running speeds, but may also lead to greater lower limb vertical velocity at touchdown, which has been established as a critical factor for generating the large mass-specific vertical forces required for high speed running (Clark et al., 2017; Mann and Murphy, 2018). Potentially, the angular velocity of the front swing thigh as it extends during the last portion of the flight phase could be directly related to the lower limb velocity at touchdown, serving as a contributing factor to vertical force application and running speed.

Therefore, the aim of this study was to examine thigh angular motion and kinematic factors influencing vertical force application across a range of speeds. Our first hypothesis was that thigh angular velocity would demonstrate a positive and linear relationship with running speed across a range of sub-maximal and maximal intensities. Our second hypothesis was that thigh angular velocity would demonstrate a positive and linear correlation with lower limb velocity at touchdown across a range of running speeds, lending insight into the relationship between thigh angular motion and vertical force determinants. These concepts may contribute to the understanding of human running, with practical applications for training interventions aimed at enhancing running performance.

## RESULTS

### Running mechanics across entire range of speeds

**Fig. 1.**
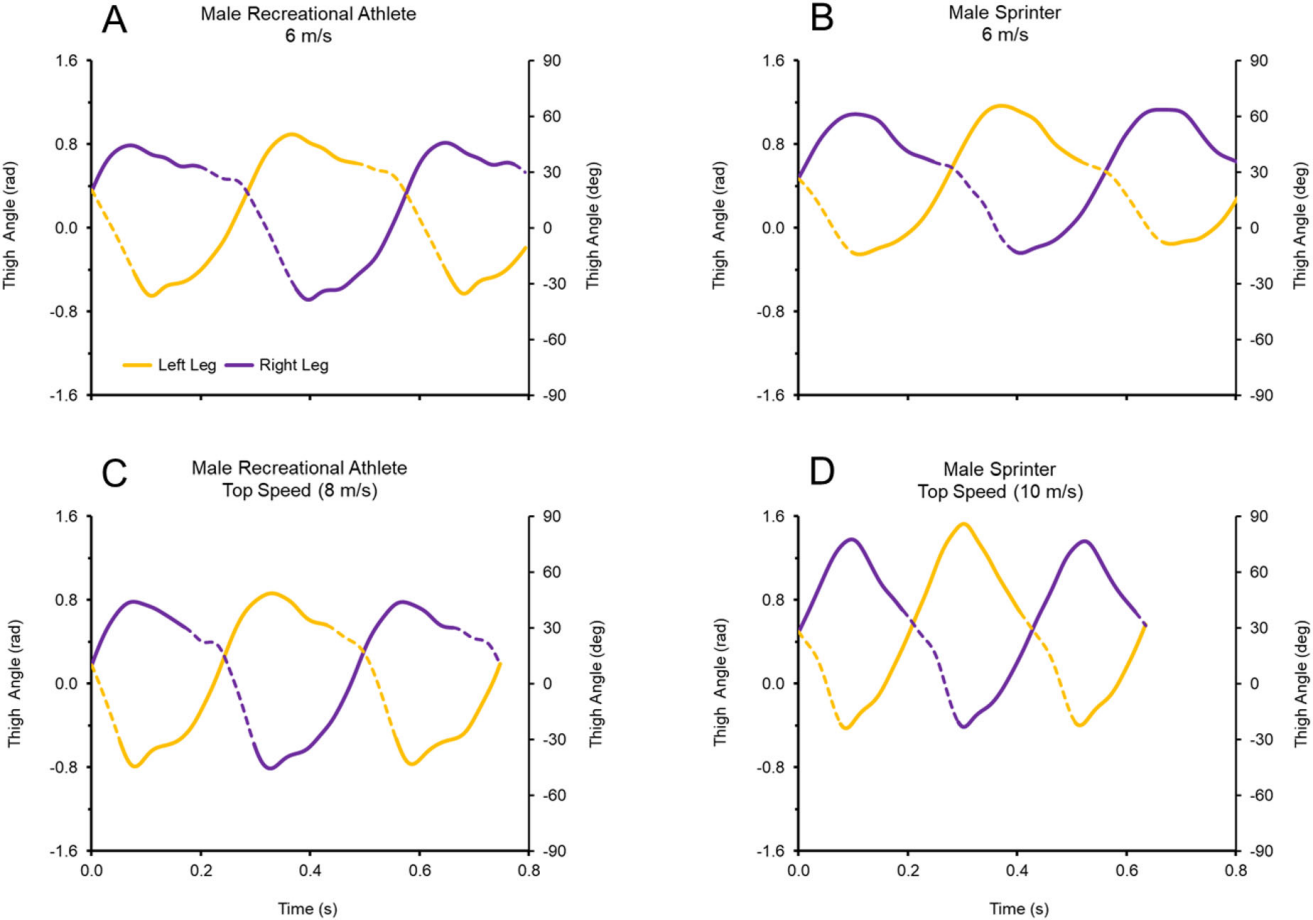
**Thigh angular position versus time for two representative subjects, with dashed regions of the graph indicating the ground contact phase.** (A,C) Male recreationally trained athlete at sub-maximal and maximal speeds. (B,D) Male sprinter at sub-maximal and maximal speeds. Faster running speeds were achieved with higher frequencies and greater total amplitudes of thigh angular motion, resulting in greater thigh angular velocities. At top speed, the slope of the angular position versus time curve during ground contact was steeper for the sprinter (D) than for the recreationally trained athlete (C), indicating a greater absolute value of the average angular velocity of the stance thigh during ground contact (ω_c_). Per Eqn 6, greater ω_c_ was a direct determinant of the faster top running speed attained by the sprinter.

The subjects differed greatly in size and athletic background, but demonstrated similar modifications to running mechanics across the range of speeds. Representative data for thigh angular position versus time is presented in Fig. 1, with a male recreationally trained athlete at sub-maximal and maximal speeds displayed in Fig. 1A and C, and a male sprinter at sub-maximal and maximal speeds displayed in Fig. 1B and D.

**Fig. 2.**
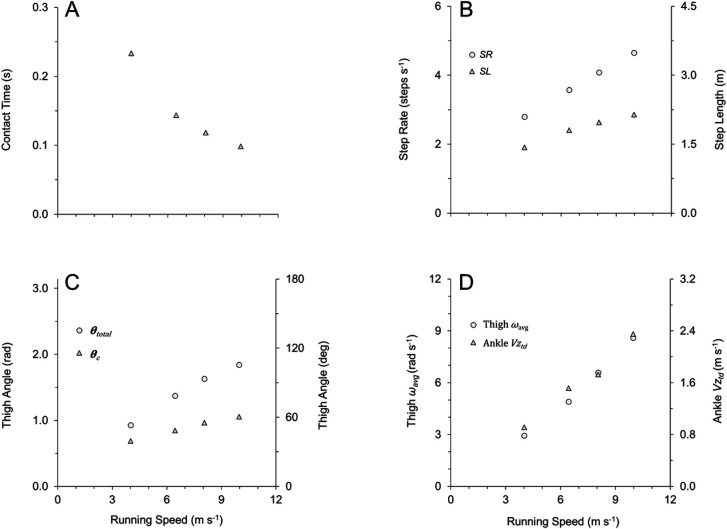
**Kinematic variables for a single representative subject (male sprinter) across his individual range of speeds.** (A) Ground contact time, T_c_. (B) Step rate, SR, and step length, SL. (C) Total thigh excursion during ground contact phase, θ_c_, and total thigh excursion from peak extension through peak flexion, θ_total_. (D) Average thigh angular velocity during entire gait cycle, ω_avg_, and lower limb vertical velocity at instant of touchdown (Ankle Vz_td_).

**Fig. 3.**
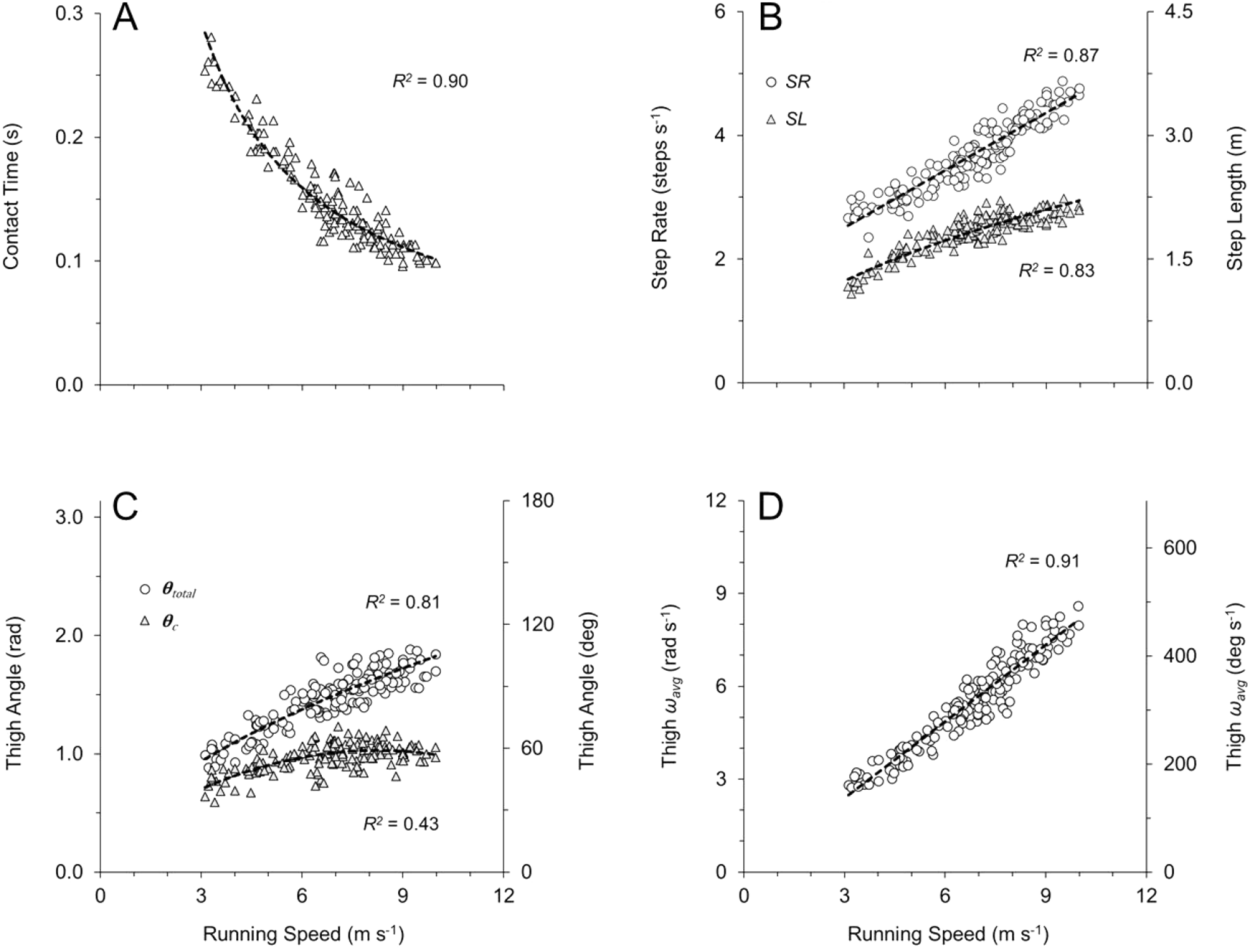
**Kinematic variables for all subjects across all speeds (*n*=154).** Best-fit equations and *P*-values listed here where appropriate, with R^2^ values presented in accompanying panels. (A) Ground contact time (T_c_=0.78*x*^−0.89^). (B) Step rate (SR=0.31*x*+1.58, *P*<0.0001) and step length (SL=0.72*x*^0.49^). (C) Total thigh excursion during ground contact phase (θ_c_=−0.01*x*^2^+0.20*x*+0.22) and total thigh excursion from peak extension through peak flexion (θ_total_=0.51*x*^0.56^). (D) Average thigh angular velocity during entire gait cycle (ω_avg_=0.82*x*−0.08, *P*<0.0001).

Spatial–temporal variables and thigh angular kinematic variables are presented for a single subject (male sprinter) across his individual range of speeds in Fig. 2, and for the subject population as a whole across all subjects and trials in Fig. 3. Spatial–temporal and thigh angular kinematic variables across all subjects and trials (categorized by slow, intermediate and fast speeds) are also presented in Table 1 and Table S2.

**Table 1.**
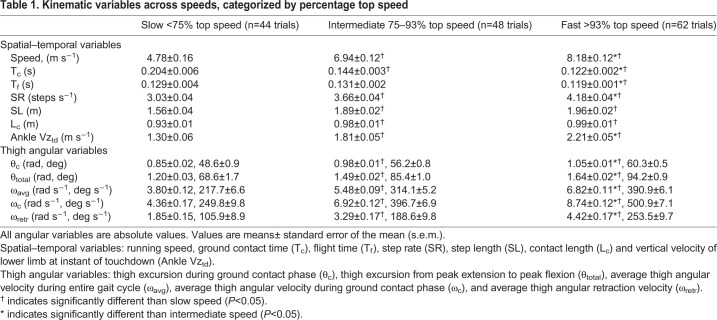
**Kinematic variables across speeds, categorized by percentage top speed**

For the spatial–temporal variables, contact time (T_c_) decreased with increasing speed for individual subjects (Fig. 2A) and for all subjects and trials [Fig. 3A; Table 1: *F*(2,151)=135.1, *P*<0.001]. Flight time (T_f_) was slightly briefer in duration at fast speeds than at slow and intermediate speeds for all subjects and trials [Table 1: *F*(2,151)=7.9, *P*<0.001], although the differences in T_f_ between slow and intermediate speeds were not statistically significant (Table 1: *P*>0.8). Step rate (SR) increased across the range of speeds for both individual subjects (Fig. 2B) and for all subjects and trials [Fig. 3B; Table 1: *F*(2,151)=179.4, *P*<0.0001]. Step length (SL) increased across speeds for individual subjects (Fig. 2B) and for all subjects and trials [Fig. 3B; Table 1: *F*(2,151)=67.1, *P*<0.0001], although the increases in SL from intermediate to fast speeds were not statistically significant (Table 1: *P*=0.17). Across all subjects and trials, the mean distance travelled by the COM during ground contact (L_c_) was 0.97±0.01 m. However, L_c_ increased slightly across speeds (Table 1: *H*=10.4, *P*<0.01), though the increases in L_c_ from intermediate to fast speeds were not statistically significant (Table 1: *P*>0.9). Analysis regarding vertical velocity of the lower limb (ankle marker) at the instant of touchdown (Ankle Vz_td_) is presented in the last section of the Results.

With respect to thigh angular kinematics, faster speeds were generally characterized by higher frequency and greater total amplitude of thigh angular motion (θ_total_), as illustrated by representative data in Fig. 1. Increases in θ_total_ occurred with speed for individual subjects (Fig. 2C) and for all subjects and trials (Fig. 3C; Table 1: *H*=91.6, *P*<0.0001). Increases in θ_total_ across the range of speeds were due to both increases in thigh flexion (θ_flex_) and increases in thigh extension (θ_ext_) (Appendix B and Table S2). Across all subjects and trials, the total excursion angle during contact (θ_c_) approached one radian (mean θ_c_=0.97±0.01 radian). However, there were increases in θ_c_ across the range of speeds for individual subjects (Fig. 2C) and for all subjects and trials (Fig. 3C; Table 1: *H*=71.8, *P*<0.0001). Increases in θ_c_ across the range of speeds were due to both increases in thigh touchdown (θ_td_) and in thigh takeoff (θ_to_) (Appendix B and Table S2).

All measures of thigh angular velocity significantly increased across speeds. There was a positive and linear relationship between absolute average thigh angular velocity during the entire gait cycle (ω_avg_) and speed for individual subjects (Fig. 2D) and for all subjects and trials [Fig. 3D; Table 1: *F*(2,151)=204.2, *P*<0.0001]. Additionally, the average angular velocity of the stance thigh during ground contact (ω_c_) increased with speed for all subjects and trials [ω_c_=1.14*x*−0.89, *R*^2^=0.88, *P*<0.0001; Table 1: *F*(2,151)=257.4, *P*<0.0001]. Per Eqn 4, T_c_ showed a strong relationship with ω_c_ for all subjects and trials (T_c_=0.002*x*^2^−0.045*x*+0.36 where *x* is ω_c_, *R*^2^=0.91), and T_c_ also had a strong relationship with ω_avg_ for all subjects and trials (T_c_=0.62*x*^−0.86^ where *x* is ω_avg_, *R*^2^=0.90). Per Eqn 6, ω_c_× leg length (L_0_) showed a direct positive linear relationship with speed for all subjects and trials with the best fit line constrained through the origin (ω_c_×L_0_=0.94*x*, *R*^2^=0.91, *P*<0.0001). Also, ω_retr_ increased with speed across all subjects and trials (ω_retr_=0.80*x*−2.14, *R^2^*=0.71, *P*<0.0001; Table 1: *H*=70.2, *P*<0.0001). Finally, across all subjects and trials, ω_avg_ was related to the other two measures of thigh angular velocity, as ω_avg_ had a strong positive relationship to both ω_c_ (ω_c_=1.35*x*−0.55 where *x* is ω_avg_, *R*^2^=0.92, *P*<0.0001) and ω_retr_ (ω_retr_=0.98*x*−2.10 where *x* is ω_avg_, *R^2^*=0.80, *P*<0.0001).

### Running mechanics across top speeds

**Fig. 4.**
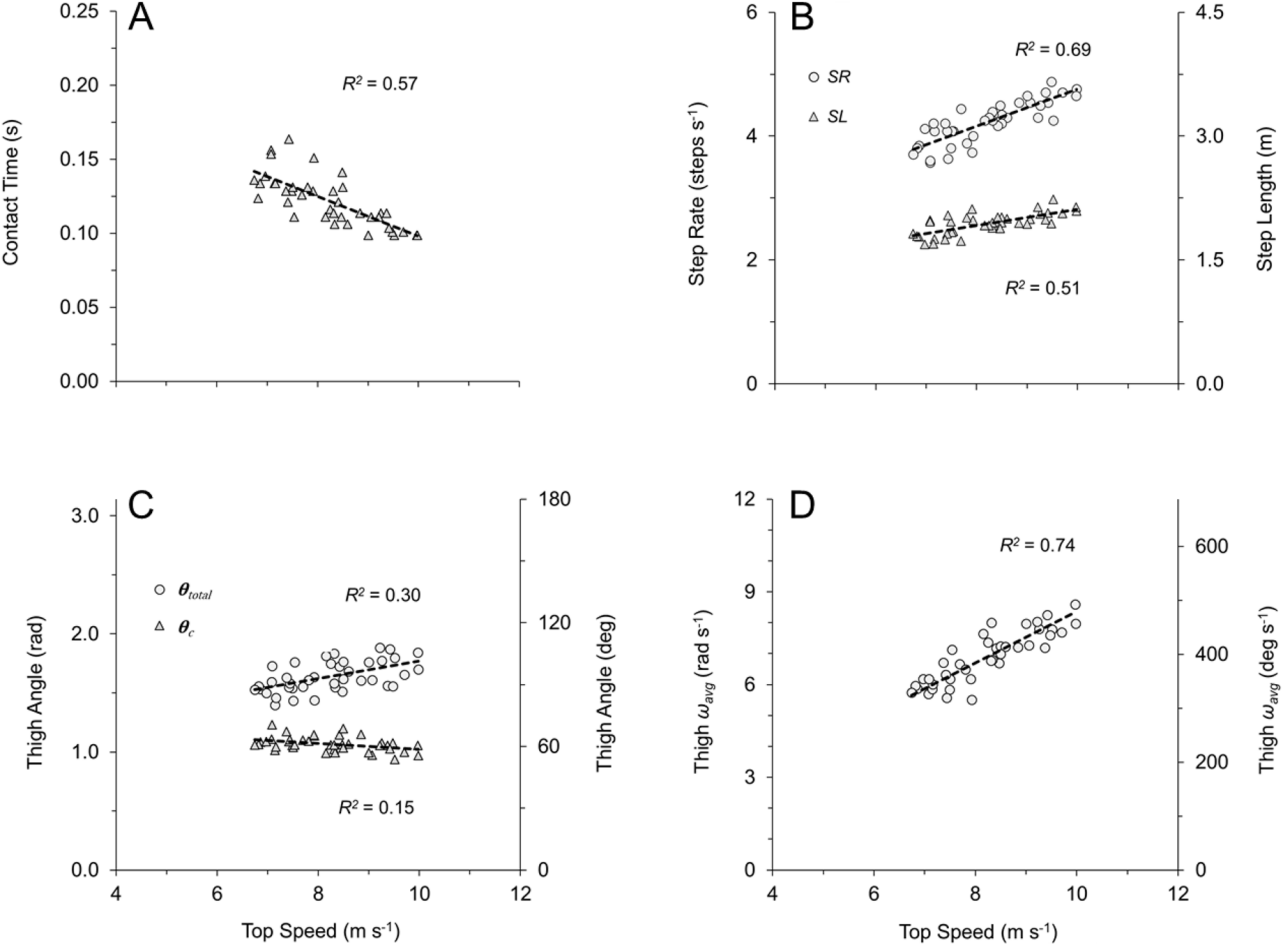
**Kinematic variables for all subjects across top speed trials (*n*=40).** Best-fit equations and *P*-values listed here, with R^2^ values presented in accompanying panels. (A) Ground contact time (T_c_=−0.013*x*+0.23, *P*<0.001). (B) Step rate (SR=0.30*x*+1.78, *P*<0.0001) and step length (SL=0.10*x*+1.14, *P*<0.0001). (C) Total thigh excursion during ground contact phase (θ_c_=−0.03*x*+1.28, *P*=0.014) and total thigh excursion from peak extension through peak flexion (θ_total_=0.07*x*+1.03, *P*<0.001). (D) Average thigh angular velocity during entire gait cycle (ω_avg_=0.77*x*+0.58, *P*<0.0001).

Kinematic variables were analyzed across all top speed trials and with runners grouped by top speed and sex. There were significant differences in top speed for the fast versus slow subjects when analyzed within sex (Table 2, males: *P*<0.001, Δ=14.2%; females: *P*<0.01, Δ=13.2%). Faster top speeds were significantly related to briefer T_c_ across all top speed trials (Fig. 4A) and when analyzing fast versus slow runners within sex (Table 2, males: *P*<0.001, Δ=21.3%; females: *P*<0.01, Δ=15.0%). Faster top speeds were also significantly related to greater SR across the entire subject population (Fig. 4B) and when analyzing fast versus slow runners within sex (Table 2, males: *P*<0.01, Δ=8.6%; females: *P*=0.04, Δ=6.5%). Similar relationships were found for SL across all top speed trials (Fig. 4B) and when analyzing fast versus slow runners within sex (Table 2, males: *P*=0.002, Δ=5.7%; females: *P*=0.015, Δ=6.6%). However, top speed was not significantly related to T_f_ (across all top speed trials: T_f_=−0.03*x*+0.14, *R*^2^=0.07, *P*=0.10; Table 2, males: *P*=0.30, Δ=4.3%; females: *P*=0.60, Δ=2.5%) or L_c_, (across all top speeds L_c_=0.01*x*+0.93, *R*^2^=0.01, *P*=0.62; Table 2, males: *P*=0.07, Δ=6.8%; females: *P*=0.51, Δ=2.2%).

**Table 2.**
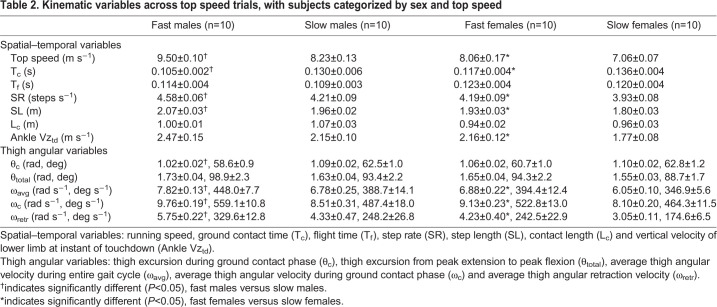
**Kinematic variables across top speed trials, with subjects categorized by sex and top speed**

For thigh angular kinematics, faster top speeds were characterized by higher frequency and greater θ_total_ (see Fig. 1C versus D for representative data). Across all top speed trials, θ_total_ was positively and significantly related to faster top speeds (Fig. 4C), although the differences in θ_total_ did not reach significance when analyzing fast versus slow runners within sex (Table 2, males: *P*=0.099, Δ=5.7%; females: *P*=0.057, Δ=6.2%). For all top speed trials, θ_c_ was slightly greater than one radian (mean θ_c_=1.07±0.01 radian). However, θ_c_ was negatively and significantly related with speed across all top speed trials (Fig. 4C), and differences in θ_c_ were significant for males (but not females) when analyzing fast versus slow runners within sex (Table 2, males: *P*=0.009, Δ=6.6%; females: *P*=0.057, Δ=3.4%). Additional top speed data for θ_td_, θ_to_, θ_ext_ and θ_flex_ are presented in Appendix B and Table S3.

Across all top speed trials, ω_avg_ was positively and significantly related to faster top speeds (Fig. 4D) and when analyzing fast versus slow runners within sex (Table 2, males: *P*=0.002, Δ=14.2%; females: *P*=0.003, Δ=12.8%). Likewise, ω_c_ was positively and significantly related to faster top speeds across all top speed trials (ω*_c_*=0.77*x*+2.57, *R*^2^=0.58, *P*<0.0001) and when analyzing fast versus slow runners within sex (Table 2, males: *P*=0.003, Δ=13.7%; females: *P*=0.003, Δ=11.9%). Finally, ω_retr_ was positively and significantly related to faster top speeds across all top speed trials (ω_retr_=1.18*x−*5.36, *R*^2^=0.64, *P*<0.0001) and when analyzing fast versus slow runners within sex (Table 2, males: *P*=0.014, Δ=28.2%; females: *P*=0.011, Δ=32.5%).

### Lower limb impact velocity, running speed, and thigh angular kinematics

**Fig. 5.**
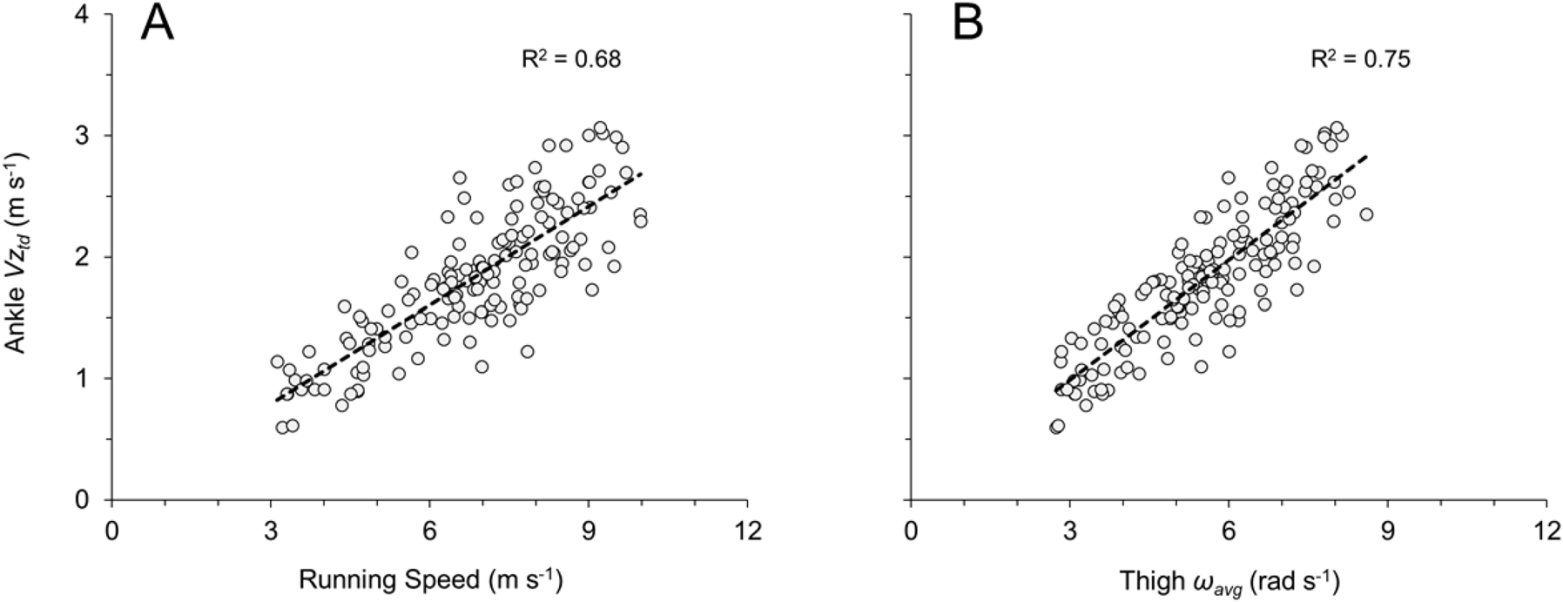
**Lower limb vertical velocity at instant of touchdown (Ankle Vz_td_) for all subjects and trials (*n*=154).** Best-fit equations and *P*-values listed here, with R^2^ values presented in accompanying panels. (A) Ankle Vz_td_ versus running speed (Ankle Vz_td_=0.27*x*−0.02, *P*<0.0001). (B) Ankle Vz_td_ versus average thigh angular velocity during entire gait cycle (Ankle Vz_td_=0.33*x*+0.01 where *x* is ω_avg_, *P*<0.001).

Ankle Vz_td_ demonstrated positive and linear relationships with both running speed and with measures of thigh angular velocity (ω_avg_ and ω_retr_). Ankle Vz_td_ increased across the range of speeds for individual subjects (Fig. 2D) and for all subjects and trials (Fig. 5A; Table 1: *H*=72.1, *P*<0.0001). Faster top speeds were positively and significantly related to greater Ankle Vz_td_ across all top speed trials (Ankle Vz_td_=0.29*x*−0.23, *R*^2^=0.40, *P*<0.001), although when analyzing fast versus slow top speeds within sex, Ankle Vz_td_ demonstrated significant differences for females but did not reach significance for males (Table 2, males: *P*=0.118, Δ=13.9%; females: *P*=0.015, Δ=19.8%). Ankle Vz_td_ was positively and significantly related to ω_avg_ across all subjects and trials (Fig. 5B), and when analyzed for top speed trials only (Ankle Vz_td_=0.37*x*−0.41 where *x* is ω_avg_, *R*^2^=0.52, *P*<0.001). Finally, Ankle Vz_td_ was significantly related to ω_retr_ across all subjects and trials (Ankle Vz_td_=0.30*x*+0.83 where *x* is ω_retr_, *R*^2^=0.75, *P*<0.0001), and when analyzed for top speed trials only (Ankle Vz_td_=0.23*x*+1.15 where *x* is ω_retr_, *R*^2^=0.54, *P*<0.001).

## DISCUSSION

### Experimental tests of hypotheses

We undertook this investigation to explore the relationship between thigh angular motion, ground contact mechanics and running speed. Based on our framework, we hypothesized that thigh angular velocity would have a strong positive linear relationship with running speed across the entire range of trials, and across the subjects’ range of top speeds. We also hypothesized that thigh angular velocity would have a strong positive linear relationship with lower limb vertical velocity at touchdown. We recruited a heterogenous group of 40 subjects that included males and females across a range of different sizes (height range: 1.52–1.94 m, mass range: 45.5–117.0 kg), from a variety of athletic backgrounds (recreationally trained individuals, intercollegiate team sport athletes, and competitive track and field athletes), and had them run across more than a threefold range of speeds (3.1–10.0 m s^−1^).

Individual subjects ran faster by increasing both the frequency and amplitude of thigh angular motion, resulting in an increase in thigh angular velocity (Figs 1 and 2). This same pattern was consistent across all subjects and trials, as increases in running speed demonstrated a strong positive relationship with thigh angular velocity (Table 1). This was best represented by the 91% shared variance between running speed and ω_avg_ across all trials (Fig. 3D). Additionally, across all top speed trials, faster top speeds had a positive, significant relationship to both ω_avg_, ω_retr_ and ω_c_ (Table 2), with 74% shared variance between running speed and ω_avg_ across top speed trials (Fig. 4D). When top speed trials were analyzed with subjects grouped by speed and sex, all measures of thigh angular velocity were significantly greater in fast subjects compared to their slow counterparts. Comparing fast and slow runners within sex (Table 2), the percentage differences in ω_avg_ (∼13 to 14%) were nearly identical to the differences in top speed (∼13 to 14%).

Likewise, our data also supported the second hypothesis. Across a range of speeds from a slow jog to a maximal sprint, Ankle Vz_td_ demonstrated a significant positive relationship with speed (Table 1), with 68% shared variance between running speed and Ankle Vz_td_ (Fig. 5A). Similar results were evident across all top speed trials, as Ankle Vz_td_ was significantly related to top speed for the entire subject population. When comparing within speed and sex, Ankle Vz_td_ was significantly greater for fast females than slow females (Table 2), and although this differential did not reach significance for males, both fast males and fast females had Ankle Vz_td_ that were more than 13% greater than their slow counterparts. Ankle Vz_td_ was also significantly related to thigh angular velocity (ω_avg_, and ω_retr_) across the range of speeds, with 75% shared variance between Ankle Vz_td_ and ω_avg_ (Fig. 5B) and between Ankle Vz_td_ and ω_retr_. Similar findings were also evident across top speed trials, as Ankle Vz_td_ was significantly related to both ω_avg_ and ω_retr_ at top speed.

### Framework evaluation

While the data strongly supported the hypotheses, it is important to note that alternative outcomes were possible. A strong positive linear relationship between running speed and ω_avg_ may not have occurred if the fundamental framework assumptions regarding ground contact geometry (θ_c_ and L_c_) were violated. Based on prior evidence it was assumed that θ_c_≈1.0 radian and that L_c_≈L_0_≈1.0 m (Clark and Weyand, 2014; Farley et al., 1993; Gatesy and Biewener, 1991; He et al., 1991; Weyand et al., 2010). In this study, θ_c_ increased slightly across speeds for all subjects and trials (Figs 2C, 3C, and Table 1), but the mean increase in θ_c_ from intermediate to fast speeds was only 0.07 radians (∼4°). Likewise, L_c_ increased slightly across speeds (Table 1), although increases in L_c_ from intermediate to fast speeds were not statistically significant. Not surprisingly, there was some between-subject variability in L_c_, with values differing between the shortest female and the tallest male by more than 0.2 m at top speed. However, when normalized to L_0_, these values did not demonstrate extreme deviation from the geometric assumptions, with L_c_*/*L_0_ ratios of 1.03 and 1.11 at top speed for the shortest female and tallest male, respectively. Given that the mean θ_c_ was 0.97±0.01 radians across all subjects and trials, and the mean L_c_ across all subjects and trials was 0.97±0.01 m, the experimental data generally adhered to the assumptions presented in the framework. Prior research has suggested that humans and other bipedal runners are likely constrained to these excursion angles and contact lengths due to leg extensor muscle mechanical advantage (Biewener, 1989; Weyand et al., 2010).

A second assumption related to symmetrical thigh flexion and extension values about the zero-axis during both ground contact and flight phases. Our assumption of symmetrical θ_td_ and θ_to_ angles of 0.5 radians during ground contact was not entirely supported, although mean θ_td_ and θ_to_ values approached 0.5 radians at intermediate and faster speeds (Appendix B and Table S2). Conceivably, if the θ_td_ angles had deviated further from the expected values, it could have weakened the correlation between thigh angular velocity (ω_avg_ and ω_retr_) and Ankle Vz_td_. As it related to thigh flexion and extension during flight, recent evidence (Mann and Murphy, 2018) has suggested that runners might exhibit more ‘front-side’ mechanics at faster speeds (i.e., more thigh flexion and less thigh extension during the flight phase). This evidence questions the veracity of assuming symmetrical flexion/extension about the zero-axis during the flight phase. In our data set, θ_total_ increased across the range of speeds due to increases in θ_flex_ that were proportionately bigger than the increases in θ_ext_ ( Appendix B and Table S2). Furthermore, across the top speed trials, fast males and fast females had θ_total_ that was shifted more front-side, with a greater proportion of θ_total_ occurring in θ_flex_ than in θ_ext_, compared to slow males and slow females (Fig. 1C,D, Appendix B and Table S3).

Finally, the thigh segment motion was qualitatively oscillatory and reciprocal. Peak flexion of one thigh generally exhibited temporal coordination with peak extension of the other thigh, especially at faster speeds (Fig. 1). Although the data exhibited some deviation from exact sinusoidal motion, this was expected due to slightly variable coordination strategies across a broad range of speeds. This variation was not substantial enough to disrupt another important assumption related to continuous harmonic thigh motion throughout the gait cycle, which indicates that ω_avg_ must increase for ω_c_ to increase. Our data supported this premise, as across all subjects and trials, there was 92% shared variance between ω_avg_ and ω_c_, and 90–91% shared variance between T_c_ and measures of thigh angular velocity (ω_avg_ and ω_c_). Therefore, despite some data not fully conforming to simplifying assumptions, the two experimental hypotheses were still supported strongly by the results.

### Building on prior research

**Fig. 6.**
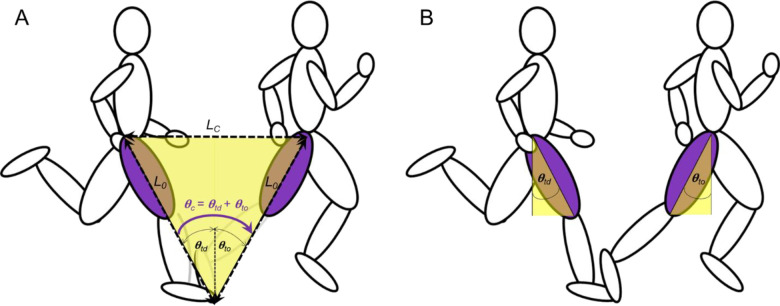
**Simplified planar representation of the ground contact phase.** (A) Geometry of the ground contact leg, including: symmetrical θ_td_ and θ_to_, θ_c_, L_0_ and L_c_. (B) θ_td_ and θ_to_ during the ground contact phase. This framework assumes that the leg angle (from ball of foot to hip, θ_td_ or θ_to_ in A) is equal to the thigh angle (θ_td_ or θ_to_ in B, respectively).

**Fig. 7.**
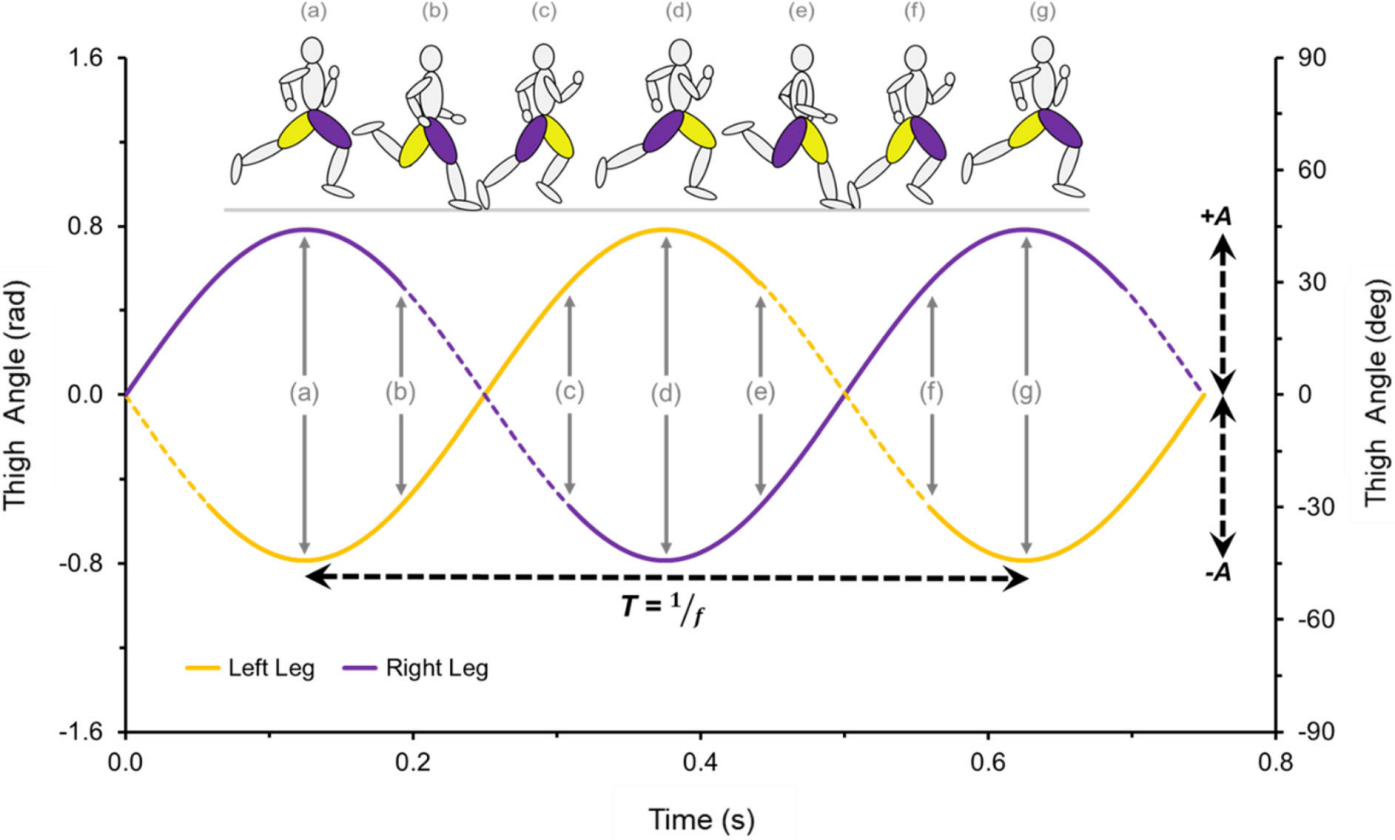
**Simplified representation of thigh angular motion during the entire gait cycle.** This representation assumes symmetrical anti-phase flexion and extension values during ground contact and flight phases. Thigh angular kinematics as a function of time are determined by the parameters of frequency (*f*=1/T, where T is the period) and amplitude (A). The figures in inset diagrams a–g illustrate the thigh angular position in correspondence with the angular motion presented in the graph. The dashed regions of the graph indicate the ground contact phase, with ω_c_ corresponding to the slope of the angular position versus time curve from touchdown (+0.5 radian) to takeoff (−0.5 radian).

**Fig. 8.**
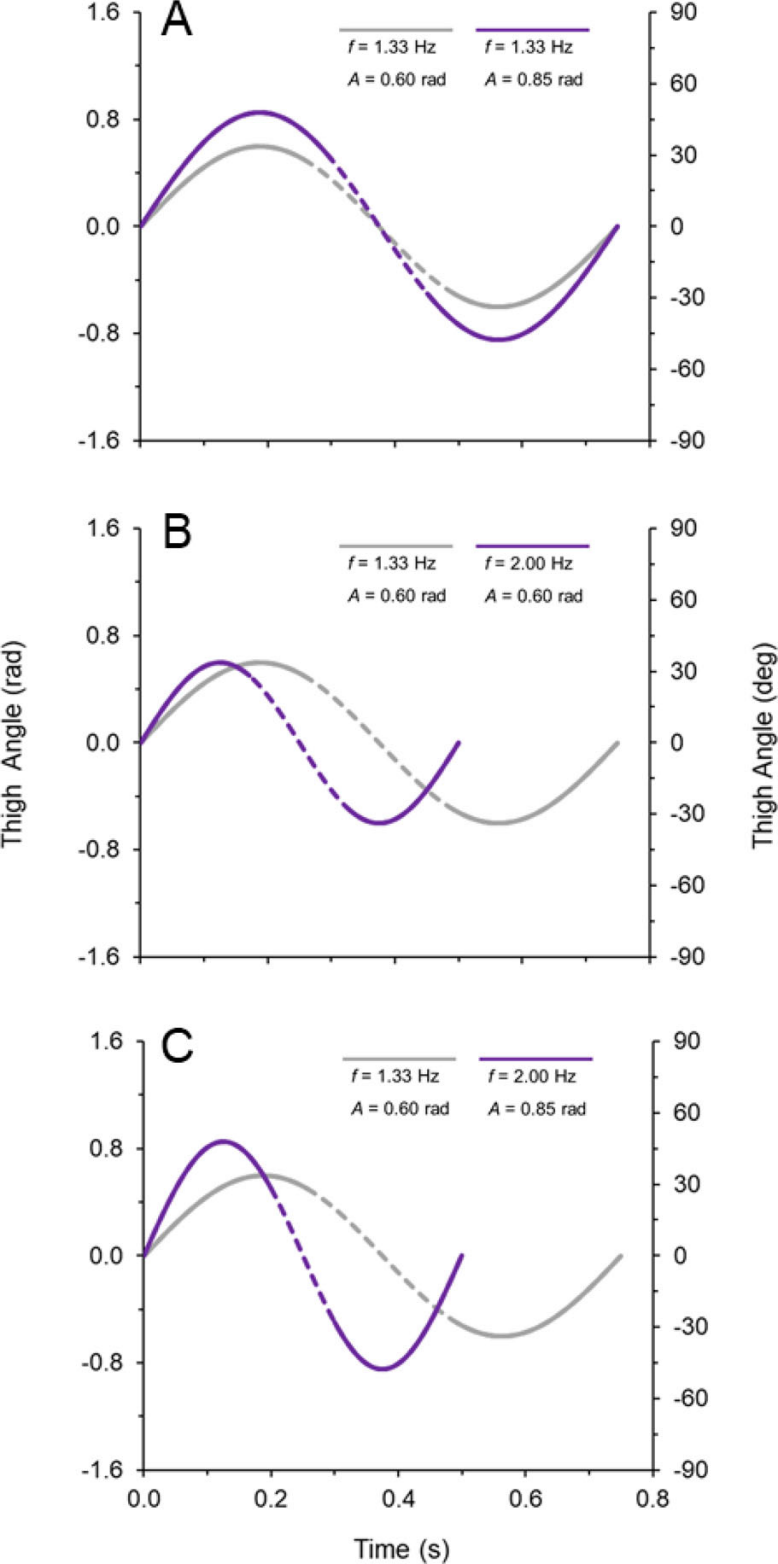
**Example graphs of thigh angular position versus time for one limb, examining the theoretical effects of altering frequency (*f*=1/T) and amplitude (A).** In A–C, the gray line has *f*=1.33 Hz and *A*=0.60 radians. The purple line illustrates the effects on thigh angular velocity that result from altering frequency and amplitude. (A) Increasing A without altering *f*. (B) Increasing *f* without altering A*.* (C) Increasing both A and *f*. The dashed regions of the graph indicate the ground contact phase, with average ω_c_ corresponding to the slope of the angular position versus time curve. In all three panels, the purple line has greater ω_c_ than the gray line because of increased A and/or *f*.

Several previous publications have observed the relationship between measures of leg angular velocity and running speed (Kivi et al., 2002; Kunz and Kaufman, 1981; Mann and Herman, 1985; Mann and Murphy, 2018; Miyashiro et al., 2019). Furthermore, faster running speeds have been associated with measures of hip joint strength, power, torque, work and muscle activation (Belli et al., 2002; Bezodis et al., 2008; Copaver et al., 2012; Deane et al., 2005; Dorn et al., 2012; Nagahara et al., 2020; Schache et al., 2011). Here, Figs 6–8 and Eqns 1–8 (Materials and Methods) provide a geometric/mathematical explanation for the linear relationship between ω_avg_ and running speed. Data from this investigation supports these concepts, as results indicated a direct positive linear relationship between increases in ω_avg_ of 1.0 rad s^−1^ and increases in running speed of ∼1.2 m s^−1^ (Figs 3D and 4D) and Ankle Vz_td_ of ∼0.33 m s^−1^ (Fig. 5B).

Contributing to the strong correlations was the measurement of ω_avg_, and not just thigh angular velocity in one specific phase of the cycle such as flexion or extension. Measuring ω_avg_ provided a comprehensive representation of the oscillatory frequency and amplitude necessary to achieve a given running speed. Although thigh angular acceleration was not directly reported in this study, the measurement of ω_avg_ over the entire gait cycle accounted for the rapid angular accelerations that occurred as the thigh reversed from flexion to extension or vice versa. These vigorous flexion/extension reversals were more pronounced in faster runners than slower runners at top speed (Fig. 1C,D), and undoubtedly contributed to the greater ω_avg_ observed in fast runners than their slow counterparts.

The results presented here also provide insight into the limb kinematics underlying force application. Numerous prior investigations have established the relationship between high-speed running and mass-specific vertical force (Bundle and Weyand, 2012; Clark and Weyand, 2014; Dorn et al., 2012; Kuitunen et al., 2002; McGowan et al., 2012; Weyand et al., 2000, 2010), with recent research indicating that greater vertical forces are due in large part to faster vertical velocities of the lower limb at touchdown combined with a rapid deceleration of the lower limb during initial ground contact (Clark et al., 2014, 2017). In the present study, the values of Ankle Vz_td_ across a range of speeds (Fig. 5A) closely aligned with data from recent publications (Clark et al., 2017; Udofa et al., 2019). Additionally, although correlation and causation must be interpreted with caution, our results suggest that increased thigh angular velocities contribute to the greater Ankle Vz_td_ observed at faster speeds (Fig. 5B).

Collectively, these findings suggest that thigh angular velocity is related to vertical force determinants. This is perhaps best illustrated in Fig. 2D, which depicts nearly parallel increases in ω_avg_ and Ankle Vz_td_ across the subject's range of speeds. Slower speeds are characterized by reduced thigh oscillatory frequencies and amplitudes, resulting in relatively decreased thigh angular velocities and slower vertical velocities of the lower limb at touchdown. Faster running speeds require increased thigh oscillatory frequencies and amplitudes, resulting in greater thigh angular velocities and faster vertical velocities of the lower limb at touchdown. The latter, when combined with a stiff ground contact and rapid lower limb deceleration upon ground contact (Clark et al., 2017), contributes to the greater mass-specific vertical forces required for faster running speeds (Kuitunen et al., 2002; McGowan et al., 2012; Weyand et al., 2000).

### Future considerations and practical applications

While building on prior findings, this study raises many topics worthy of further investigation. First, we intentionally recruited a heterogenous group of male and female subjects from a range of sizes and athletic backgrounds, and analyzed running mechanics across a threefold range of speeds. Undoubtedly, some of the strong statistical relationships we found between speed and the kinematic variables were due to this heterogenous subject pool and broad range of speeds. However, we justified our approach based on the desire to elucidate macro-level determinants of running speed from slow jogging to maximal sprinting. Although further research is necessary to confirm whether the relationships established here will generalize to a less diverse group of athletes within more narrow ranges of speed (i.e. elite sprinters at top speed), the existing evidence regarding the thigh angular kinematic determinants of speed (Mann and Murphy, 2018) suggest that our major findings may generalize to homogenous subject populations.

Potential applications of these findings for performance improvement in human sprinting are intriguing. Coaching cues such as ‘whip from the hip’ have been popularized by some well-known practitioners, emphasizing a vigorous scissor-like action of the thighs (Bosch and Klomp, 2005), and these instructions appear to have practical merit. Similar to a hammer striking a nail, high-speed running requires fast rotational and tangential velocity prior to impact combined with a stiff collision upon impact. Likewise, our findings suggest that greater top speeds require fast thigh angular retraction velocities in an open kinetic-chain movement prior to ground contact, combined with a stiff stance limb that allows the thigh to extend rapidly in a closed kinetic chain movement throughout ground contact. We speculate that a relatively stiff lower limb during ground contact is not only imperative for brief contact times and large vertical forces (Arampatzis et al., 1999; Belli et al., 2002; Clark et al., 2017; Kuitunen et al., 2002), but also to allow the thigh to continuously extend at high angular velocities throughout ground contact. Any excessive compliance in the lower limb during contact may hinder rates of thigh extension, prolonging ground contact time and decreasing running speed.

Our findings align with recent research linking faster running speeds to increased hip joint muscular activation, torque, work and power (Belli et al., 2002; Bezodis et al., 2008; Copaver et al., 2012; Dorn et al., 2012; Nagahara et al., 2020; Schache et al., 2011). However, few studies have examined methods for longitudinal improvement of these determinants. Although the effects of lower body wearable resistance (Macadam et al., 2017) and hip flexor strengthening (Deane et al., 2005) have been investigated, the best methods for enhancing thigh angular velocity during sprinting are not clearly established, and require expanded investigation. Conceivably, coaches and athletes could aim to enhance thigh flexion angular velocity in open-kinetic chain movements (forward swing phase), or thigh extension angular velocity in open-kinetic chain (retraction) or closed-kinetic chain (ground contact) movements. Given the coordinated and reciprocal oscillatory motion generally demonstrated by the thigh segments, any longitudinal improvement in thigh extension velocity should require corresponding improvement in thigh flexion velocity, and vice versa. In other words, increasing only thigh flexion or thigh extension capability, without concomitant improvement in the other variable, is likely to limit the runner because the thighs must complete the powerful scissor-like action in synchrony. Therefore, optimal training interventions likely need to target enhancing thigh angular velocity in both flexion and extension actions during both open- and closed-kinetic chain movements.

### Concluding remarks

Here we investigated thigh motion and lower limb vertical velocity at touchdown across a broad range of runners and speeds. As hypothesized, thigh angular velocity had a direct linear relationship to both running speed and the lower limb kinematics underlying vertical force application. Our results suggest that increases in thigh angular velocity are not only necessary to match the kinematic demands for high-speed running, but also contribute to the larger mass-specific vertical forces necessary to support faster speeds. Therefore, interventions aimed at improving running performance likely need to elicit an increase in thigh angular velocity through all phases of the gait cycle.

## MATERIALS AND METHODS

### Framework

A simplified planar representation of the ground contact phase is depicted in Fig. 6. This framework assumes symmetrical touchdown and takeoff angles during ground contact and that the leg angle (from ball of foot to hip, θ_td_ or θ_to_ in Fig. 6A) is equal to the thigh angle (θ_td_ or θ_to_ in Fig. 6B, respectively). The L_c_ is determined by leg length L_0_ and θ_c_:(1)
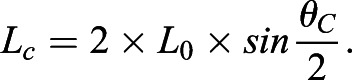
For faster running speeds, prior research has suggested that the total excursion angle during contact is approximately 1.0 radian for humans and other bipedal runners (Farley et al., 1993; Gatesy and Biewener, 1991; He et al., 1991). Thus, for normal contact excursion angles where θ_c_≈1.0 radian, Eqn 1 demonstrates that contact length is approximately equal to leg length (L_c_≈L_0_). Furthermore, since horizontal velocity during the flight phase is constant (negating wind resistance), the runner's forward speed is determined by the time it takes the COM to traverse L_c_, where T_c_ is ground contact time (Weyand et al., 2010):(2)
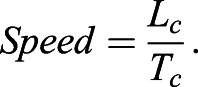
Because L_c_≈L_0_, and this distance is generally limited to about 1.0 m for most adult human runners at faster running speeds (Clark and Weyand, 2014; Weyand et al., 2010), increases in speed are usually accompanied by decreases in T_c_ (Dorn et al., 2012; Nummela et al., 2007; Weyand et al., 2010).

Additionally, leg angular velocity is a crucial determinant of locomotor stability and control (Seyfarth et al., 2003). Average angular velocity of the stance thigh during ground contact (ω_c_) is equal to the total contact excursion angle divided by ground contact time:(3)
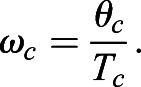
Eqn 3 can be rearranged so that T_c_ is expressed as a function of θ_c_ and ω_c_:(4)
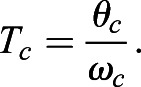
Inserting Eqn 4 into Eqn 2 and substituting L_o_ for L_c_ yields Eqn 5:(5)
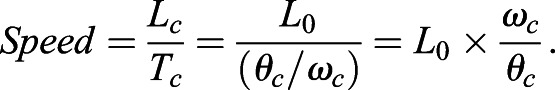
Assuming a total excursion angle θ_c_ equal to 1.0 radian, running speed is directly related to L_0_ and ω_c_:(6)
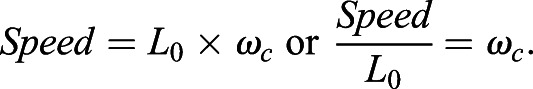
Furthermore, harmonic oscillatory thigh motion has been observed during high-speed running in humans (Novacheck, 1998; Sides, 2015), with contralateral limbs exhibiting reciprocal anti-phase flexion and extension during bipedal gait (Blum et al., 2014). Fig. 7 depicts a simplified example of thigh angular motion that assumes symmetrical anti-phase flexion and extension values during ground contact and flight phases. Because this framework is based on equations of harmonic motion, thigh angular kinematics as a function of time are determined by the parameters of frequency (*f*=1/T, where T is the time period) and amplitude (A). Thigh angular position as a function of time is displayed in Eqn 7:(7)
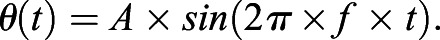
Thigh angular velocity as a function of time is displayed in Eqn 8:(8)
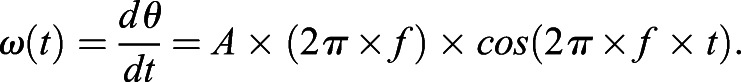
Graphically, the ground contact phase is represented by the dashed lines in Fig. 7, from touchdown (+0.5 radian) to takeoff (-0.5 radian). Thus, ω_c_ is equal to the slope of the angular position versus time curve during the ground contact phases (slope of the dashed line in Fig. 7B,C,E and F). The steeper the slope of the angular position versus time curve in Fig. 7B,C,E,F, the greater the absolute value of ω_c_, and the faster the running speed (normalizing for L_0_, per Eqn 6).

The figures and equations above indicate that thigh angular velocity must be regulated to maintain a constant forward running speed. It has been suggested that the limbs function as harmonic oscillators (McGeer, 1990), which implies that ω_c_ is related to the average of the absolute value of thigh angular velocity during ω_avg_. Because of the continuous harmonic motion of the thighs, it would be expected that a faster ω_c_ would correspond to a proportionately faster ω_avg_. From a mathematical standpoint, Eqns 7 and 8 imply that ω_c_ and ω_avg_ can be improved by increasing either *f* or A, or increasing some combination of both (see examples in Fig. 8). Perhaps more importantly, the equations above dictate that ω_c_ and ω_avg_ must both increase for running speed to increase (per hypothesis 1).

Enhanced ω_avg_ should be related to an increased lower limb vertical velocity at touchdown. As the front swinging thigh extends and causes limb retraction during the last portion of the flight phase directly prior to impact (e.g. Fig. 7A,B,D,E), faster angular velocity of the thigh should result in faster tangential and vertical velocity of the lower limb in the distal portion of the leg. Therefore, as ω_avg_ increases in proportion to running speed, this should also be associated with increases in thigh angular retraction velocity (ω_retr_) prior to touchdown, and both of these measures should be positively related to vertical velocity of the lower limb at touchdown across the range of running speeds (per hypothesis 2).

### Subjects and participation

All testing and data collection were completed in one 90 min testing session at the West Chester University of Pennsylvania laboratory and indoor athletic facility. A total of 40 subjects volunteered and provided written informed consent in accordance with the West Chester University of Pennsylvania Institutional Review Board. This included 20 males (mean±s.e.m., age: 21.6±0.5 years, height: 1.80±0.01 m, leg length: 0.94±0.01 m, mass: 79.7±3.0 kg) and 20 females (age: 21.7±0.4 years, height: 1.67±0.02 m, leg length: 0.89±0.01 m, mass: 59.0±1.4 kg). Male subjects included nine intercollegiate or post-collegiate track and field athletes [≤400 m sprints (*n*=7), horizontal jumps (*n*=2)], five intercollegiate team sport athletes [soccer (*n*=2), rugby (*n*=2), American football (*n*=1)] and six recreationally trained subjects. Female subjects included six intercollegiate or post-collegiate track and field athletes [≤400 m sprints (*n*=5), 100 m hurdles (*n*=1)], eight intercollegiate team sport athletes [gymnastics (*n*=3), soccer (*n*=2), rugby (*n*=1), softball (*n*=1), basketball (*n*=1)], and six recreationally trained subjects. Complete subject descriptive characteristics are listed in Table S1. Per inclusionary criteria, all subjects were healthy and regularly active (defined as exercising three or more times per week) at the time of testing.

### Testing procedures

After subjects reviewed and signed consent forms, experimental procedures were as follows. Subjects were provided with standardized compression clothes and running track flats (Nike Waffle Racer version nine, Beaverton, OR, USA). Subjects were measured for height using a standard measuring tape and weighed on a digital scale (Supac Model EB-8008, Shanghai, China). Subjects then performed a full-body warm up including jogging, skipping, dynamic stretches and sub-maximal sprints. Next, for the purposes of motion capture, subjects wore reflective markers placed on the heel and ball of the foot on the lateral aspect of the running shoe, as well as on the lateral aspect of the ankle, knee, hip and shoulder (lateral malleolus, lateral femoral condyle, greater trochanter and acromial process, respectively). There were 12 reflective markers total, six on each side of the body. A still frame motion capture recording of each subject standing in the center of the field of view was completed prior to the testing to serve as a reference for kinematic analyses. Measurements of leg length from greater trochanter to ground were determined from the still frame motion capture recording, with an average leg-length value determined using measurements of right and left leg.

For the experimental testing, subjects completed 40 m running trials, and data were captured and analyzed on four trials over a range of speeds. Although the subjects were not directly paced by the investigators while running, they were instructed to complete the trials at progressively faster speeds, with the last trial being maximal. All trials were completed in a running lane in an indoor athletic facility with a multi-purpose floor. The running lane was 60 m in total length, allowing the subjects 20 m to safely decelerate and stop after completing each 40 m running trial. Subjects began each trial in an upright, ‘two-point’ stance with the preferred leg forward and started at their own initiative. For the trials completed at less than maximal intensity, subjects were instructed to gradually accelerate to a cone placed at 25 m, and then to run at a constant speed from 25 m through 40 m. For the maximal effort trials (interchangeably termed ‘top speed’ trials), subjects were instructed to perform an all-out acceleration from the beginning of the sprint, and continue at full speed all the way through the finish line at the 40 m mark. Subjects were allowed complete recovery between trials.

### Data collection and analysis

Three-dimensional kinematic data were recorded using an eight-camera motion capture system collecting at 200 Hz (OptiTrack Prime 13 cameras with Motive software from NaturalPoint, Corvallis, OR, USA). The field of view captured by the motion capture system was eight meters in length, from 31–39 m in the running lane. This field of view was selected to ensure that data could be collected for one complete gait cycle for even the tallest, fastest subjects with the longest stride lengths. Prior to data collection, the capture volume was dynamically calibrated using a wand with reflective markers at known spacings. After data collection, motion capture files were exported and uploaded to Microsoft Excel, where the data were up-sampled to 1000 Hz using linear interpolation and post-filtered using a low-pass, fourth-order, zero-phase-shift Butterworth filter with a cutoff frequency of 25 Hz (Winter, 1990). Positional data from the 12 markers were used to form a seven-segment model, including foot, shank and thigh on both legs, and a head-arms-trunk segment (Winter, 1990).

In addition to measuring running speed using motion capture data (described below), running speed was verified with a radar gun (Stalker ATS II; Applied Concepts Inc., Plano, TX, USA). The radar data were collected at 47 Hz and exported to Microsoft Excel for analysis. Speed versus time data were fit to a mono-exponential equation using an iterative least-squares regression routine (Chelly and Denis, 2001; Samozino et al., 2016). Speed versus time data were integrated to determine distance versus time, and then used to calculate average speed from 31–39 m. Average running speed from 31–39 m, as determined by the radar data, showed a high level of agreement compared to the motion capture method (*R*^2^=0.99 and mean absolute error <0.15 m s^−1^, see Appendix A and Fig. S1).

With regard to collecting top speed data in the field of view from 31–39 m, prior research has illustrated that team-sport athletes approach peak speed by 30 m into a sprint (Clark et al., 2019; Mendiguchia et al., 2016). Although elite sprinters may not attain peak speed until 60 or 70 m into a 100 m dash (Krzysztof and Mero, 2013), split-time data from 100 m competitions has indicated that even the world's fastest sprinters have reached greater than 94% top speed by 30–40 m (Krzysztof and Mero, 2013). Therefore, we deemed the 31–39 m field of view appropriate to capture top speed for the heterogenous group of subjects participating in this study. To assess the possibility that faster subjects in this study were still accelerating through the motion capture field of view during top speed trials, speed versus distance data from the radar were examined to ensure that subjects ran at constant speed in the motion capture zone. Constant speed was operationally defined as changes in speed ≤0.3 m s^−1^ from 31–39 m, and all trials satisfied this criterion.

### Measurements

Based on the kinematic data, spatial–temporal and thigh angular kinematic variables were quantified for all trials. Spatial-temporal variables included: running speed, T_c_, T_f_, SR, SL, L_c_ and Ankle Vz_td_. All reported values are trial averages from both right and left limbs during one complete gait cycle.

Instantaneous calculations of COM position and velocity were quantified using the positions of the 12 reflective markers and segment information from Winter (1990). Running speed was quantified from average COM horizontal velocity in the 31–39 m field of view. With regards to determining contact and flight time (T_c_ and T_f_), all trials were reviewed using the motion capture software (NaturalPoint, Corvallis, OR, USA), with instances of touchdown and takeoff determined from visual inspection of the heel and ball marker vertical position relative to the running surface. This method was validated compared to an in-ground laboratory force plate using a separate sub-sample of subjects, and demonstrated a high level of agreement with the force plate measurements (*R*^2^=0.99 and T_c_ mean absolute error <0.004 s, see Appendix A and Fig. S2). SR was calculated as the inverse of the sum of ground contact time and flight time, or SR=1/(T_c_+T_f_). SL was calculated from running speed and step rate, or SL=Speed/SR. Per Eqn 2 and Weyand et al. (2010), L_c_ was calculated by multiplying running speed and ground contact time, or L_c_=Speed×T_c_. The absolute value of vertical velocity of the ankle marker at the instant of touchdown (Ankle Vz_td_) was used as a proxy for lower limb impact velocity (per Clark et al., 2017).

Additionally, several thigh angular kinematic variables were determined, including: thigh angular position at touchdown (θ_td_), thigh angular position at takeoff (θ_to_), total thigh excursion during the ground contact phase (θ_c_, the sum of θ_td_ and θ_to_), peak thigh extension during flight (θ_ext_), peak thigh flexion during flight (θ_flex_), total thigh excursion from peak extension through peak flexion (θ_total_, the sum of θ_ext_ and θ_flex_), average thigh angular retraction velocity measured from peak thigh flexion until touchdown ω_retr_, average thigh angular velocity during the ground contact phase ω_c_, and average thigh angular velocity during the entire gait cycle ω_avg_. Thigh angular kinematics were quantified in an absolute frame of reference, with the thigh angle compared to vertical, and not relative to trunk. Thigh angular velocities (ω_retr_, ω_c_, and ω_avg_) were determined by calculating the derivative of the thigh segment angular position versus time data, with angular velocities measured as absolute values. As with the spatial–temporal variables, reported thigh angular kinematic values were trial averages from one complete gait cycle, with trial averages determined from both right and left limbs.

### Statistical analysis

To examine the relationship between running speed and the kinematic variables of interest, the data were analyzed across all subjects and trials. Six of the 40 subjects had sub-maximal trials with motion capture data that were not usable due to marker occlusions. These trials were discarded, resulting in *n*=154 total trials included in the data analysis. Across all subjects and trials, the relationship between running speed and each of the kinematic variables was evaluated using either simple linear regression or nonlinear regression (power or second-order polynomial equations) to generate a best-fit equation and coefficient of determination (R^2^), with *x* representing running speed unless otherwise noted. Furthermore, differences in each of the kinematic variables were analyzed across speeds with trials divided into three categories based on percentage top speed, calculated for each subject as a percentage of his or her fastest trial. These categories were slow (<75% top speed, *n*=44 trials), intermediate (75 to 93%, *n*=48 trials), and fast (>93%, *n*=62 trials). The normality of data was determined using the D'Augustino and Pearson omnibus normality test or the Shapiro–Wilk test. Parametric data were analyzed using 3x1 analysis of variance (ANOVA) and Tukey’s post-hoc tests, and non-parametric data were analyzed using the Kruskal–Wallis test (*H* statistic) and Dunn's multiple comparisons tests.

In addition to the aforementioned analysis, top speed trials (*n*=40, one trial per subject) were independently analyzed as a separate sub-set of data, with simple linear regression used to determine the relationship between each kinematic variable and top speed. To provide further insight into these top speed trials, runners were grouped by sex (male and female) and top speed (the faster ten subjects versus the slower ten subjects for each sex). For each kinematic variable, separate independent *t*-tests were used to analyze fast versus slow males and fast versus slow females. This analysis was selected instead of a 2×2 ANOVA (sex by speed) because the primary comparisons of interest were kinematic differences between fast and slow runners analyzed within sex. For each data set, the normality of data was determined using the D'Augustino and Pearson omnibus normality test or the Shapiro–Wilk test. Parametric data were analyzed using unpaired two-tailed *t*-tests, and non-parametric data were analyzed using Mann–Whitney tests. For the top speed trials, absolute percentage difference was also used to express the magnitude of difference between fast versus slow group means for each variable, calculated as:
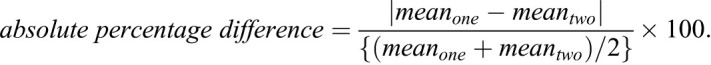
All data are expressed as mean±standard error of the mean (s.e.m.). The *a priori* threshold for all significance tests was set at *α*=0.05. Statistical analyses of thigh angular kinematics were calculated in units of radians, although for enhanced clarity, units of degrees are also presented where relevant in Figs 2–7 and in Tables 1, 2, S2, S3. Power analyses for regression and ANOVA were completed using G*Power (version 3.1.9, Kiel, Germany), based on *α*=0.05, β=0.8 and moderate effect size. All other statistics were completed using Microsoft Excel and GraphPad Prism software (version 8, San Diego, CA, USA).

## Supplementary Material

Supplementary information

## Appendix A

### Validation of running speed

Measurement of running speed using the motion capture method was verified with a radar gun (Stalker ATS II; Applied Concepts Inc., Plano, TX, USA). During all trials (*n*=154), the radar gun was placed on a tripod at a height of 1.0 m, at a distance behind the starting line of 5.0 m. Radar data were collected at 47 Hz, and then exported to Microsoft Excel for analysis. Speed versus time data were fit with a mono-exponential equation using an iterative least-squares regression routine (similar to Chelly and Denis, 2001; Samozino et al., 2016). This equation models a runner's speed versus time curve as follows:

(A 1)

where speed_max_ is the runner's maximal speed and *τ* is the time constant. Speed versus time data was then integrated to calculate distance versus time:

(A 2)

where *distance* is the horizontal position of the runner from the starting line as a function of time. After distance versus time was determined, average speed from 31–39 m was calculated. Average speed from 31–39 m, as determined by radar data, showed a high level of agreement compared to the motion capture method, with an absolute error of 0.11±0.01 m s^−1^ (mean±s.e.m.). Average speed measured by the radar gun is plotted against the motion capture method in Fig. S1.

### Validation of ground contact time

To complete data analysis, it was necessary to develop an accurate method for determining the instances of touchdown, takeoff, and total ground contact time (T_c_) using motion capture data. To accomplish this, a sub-sample of subjects completed running trials over an in-ground laboratory force plate with synchronized force and motion capture data. This sub-sample consisted of ten subjects (mean±s.e.m.: seven males, age: 24.9±2.3 years, height: 1.77±0.03 m, mass: 89.5±4.9 kg; three females, age: 21.0±0.6 years, height: 1.64±0.01 m, mass: 55.2±3.6 kg) who volunteered and provided written informed consent in accordance with the local university Institutional Review Board.

During a single 60 min testing session, subjects completed 12 trials in a 35 m laboratory runway with the force plate embedded at the 20 m point of the runway. All procedures related to subject clothing/footwear, measurements, warm-up, and motion capture marker placement were identical to those described in the Materials and Methods. Although subjects were not directly paced by the investigators while running, they were instructed to complete the trials at progressively faster speeds, with the last three trials being maximal. Subjects lined up in an upright stance 20 m behind the force plate and, at their own initiative, ran forward and stepped on the force plate during the course of a running stride. Subjects were instructed to run naturally as they ran over the force plate. If the subject missed or partially struck the force plate, or visibly altered their mechanics to strike the plate, the data obtained from the trial were discarded. A total of 120 total trials were completed, yielding 95 valid trials with synchronized force and motion capture data where the subject fully struck the force plate without altering normal running mechanics.

Data were synchronously collected with an in-ground force plate (Kistler 5691A, 0.6 m×0.9 m, Winterthur, Switzerland) and an eight-camera motion capture system (OptiTrack Prime 13, Corvallis, OR, USA). Cameras were placed around the force plate and provided a 6 m field of view centered on the force plate. Prior to data collection, the capture volume was dynamically calibrated using a wand with reflective markers at known spacings. The force plate data were collected at 1000 Hz and the motion capture data at 200 Hz. Force plate data were post-filtered in Microsoft Excel using a low-pass, fourth-order, zero-phase-shift Butterworth filter with a cutoff frequency of 25 Hz (Winter, 1990). All motion capture files were analyzed in an identical manner to that presented in the Materials and Methods. Additionally, trials were recorded using a high-speed video camera (Apple iPad 9.7, Apple USA, Cupertino, CA, USA) to ensure that the foot fully contacted the force plate and that no part of the foot landed off the force plate.

Kinematic data were analyzed to determine the spatial and temporal variables associated with touchdown and takeoff. All trials were reviewed using the motion capture software (NaturalPoint, Corvallis, OR, USA), with instances of touchdown and takeoff (and thus total T_c_) determined from visual inspection of the heel and ball marker vertical position relative to the running surface. This visual inspection method was validated compared to the force plate, which was used as the standard reference for measuring ground contact time (defined as the time when vertical force measured by the force plate exceeded 20 N). Compared to force plate data, this kinematic visual inspection method determined touchdown with a mean absolute error of 2.1±0.2 ms, takeoff with a mean absolute error of 2.7±0.2 ms, and T_c_ with a mean absolute error of 3.4±0.3 ms (mean±s.e.m.). Contact times measured by the force plate are plotted against those determined by the motion capture visual inspection method in Fig. S2.

## Appendix B

### Additional thigh angular kinematic results from all subjects and trials

Increases in θ_total_ across the range of speeds were due to both increases in θ_flex_ (θ_flex_=0.102*x*+0.273, *R*^2^=0.51, *P*<0.0001; Table S2: *H*=45.2, *P*<0.0001) and increases in θ_ext_ [θ_ext_=0.020*x*+0.367, *R*^2^=0.06, *P*=0.003; Table S2: *F*(2,151)=16.0, *P*<0.0001], though increases in θ_ext_ from intermediate to fast speeds were not significant (Table S2: *P*=0.18). Increases in θ_c_ across the range of speeds were due to both increases in θ_td_ [θ_td_=0.033*x*+0.335, *R*^2^=0.33, *P*<0.0001; Table S2: *F*(2,151)=29.5, *P*<0.0001] and increases in θ_to_ (θ_to_=0.30*x*^0.16^, *R*^2^=0.03; Table S2: *H*=13.63, *P*<0.01), though increases in θ_to_ from intermediate to fast speeds were not significant (Table S2: *P*>0.9).

### Additional thigh angular kinematic results from top speed trials

Across all top speed trials, θ_flex_ was positively and significantly related to faster top speeds (θ_flex_=0.132*x*−0.023, *R^2^*=0.38, *P*<0.0001), although differences in θ_flex_ did not reach significance when analyzing fast versus slow runners within sex (Table S3, males: *P*=0.062, Δ=16.5%; females: *P*=0.059, Δ=13.0%). Across all top speed trials, θ_ext_ was negatively and significantly related to faster top speeds (θ_ext_=−0.057*x*+1.044, *R*^2^=0.21, *P*=0.003), although differences in θ_ext_ did not reach significance when analyzing fast versus slow runners within sex (Table S3, males: *P*=0.117, Δ=16.7%; females: *P*=0.51, Δ=5.1%). Across all top speed trials, θ_td_ was not significantly related to top speed (θ_td_=0.018*x*+0.459, *R^2^*=0.05, *P*=0.159) or when analyzing fast versus slow runners within sex (Table S3, males: *P*>0.9, Δ=0.0%; females: *P*>0.9, Δ=0.1%). Across all top speed trials, θ_to_ was negatively and significantly related to faster top speeds (θ_to_=−0.043*x*+0.817, *R*^2^=0.21, *P*=0.003), although differences in θ_to_ did not reach significance when analyzing fast versus slow runners within sex (Table S3, males: *P*=0.09, Δ=15.7%; females: *P*=0.37, Δ=7.4%).

## References

[BIO053546C1] Arampatzis A., Brüggemann G.-P. and Metzler V. (1999). The effect of speed on leg stiffness and joint kinetics in human running. *J. Biomech.* 32, 1349-1353. 10.1016/S0021-9290(99)00133-5 10569714

[BIO053546C2] Belli A., Kyröläinen H. and Komi P. V. (2002). Moment and power of lower limb joints in running. *Int. J. Sports Med.* 23, 136-141. 10.1055/s-2002-20136 11842362

[BIO053546C3] Bezodis I. N., Kerwin D. G. and Salo A. I. (2008). Lower-limb mechanics during the support phase of maximum-velocity sprint running. *Med. Sci. Sports Exerc.* 40, 707-715. 10.1249/MSS.0b013e318162d162 18317373

[BIO053546C4] Biewener A. A. (1989). Scaling body support in mammals: limb posture and muscle mechanics. *Science* 245, 45-48. 10.1126/science.2740914 2740914

[BIO053546C5] Blickhan R. (1989). The spring-mass model for running and hopping. *J. Biomech.* 22, 1217-1227. 10.1016/0021-9290(89)90224-8 2625422

[BIO053546C6] Blum Y., Vejdani H. R., Birn-Jeffery A. V., Hubicki C. M., Hurst J. W. and Daley M. A. (2014). Swing-leg trajectory of running guinea fowl suggests task-level priority of force regulation rather than disturbance rejection. *PLoS ONE* 9, e100399 10.1371/journal.pone.0100399 24979750 PMC4076256

[BIO053546C7] Bobbert M. F., Schamhardt H. C. and Nigg B. M. (1991). Calculation of vertical ground reaction force estimates during running from positional data. *J. Biomech.* 24, 1095-1105. 10.1016/0021-9290(91)90002-5 1769975

[BIO053546C8] Bosch F. and Klomp R. (2005). *Running: Biomechanics and Exercise Physiology Applied in Practice*. London: Elsevier Churchill Livingstone.

[BIO053546C9] Bundle M. W. and Weyand P. G. (2012). Sprint exercise performance: does metabolic power matter? *Exerc. Sport Sci. Rev.* 40, 174-182. 10.1097/jes.0b013e318258e1c1 22732427

[BIO053546C10] Cavagna G. A. (1975). Force platforms as ergometers. *J. Appl. Physiol.* 39, 174-179. 10.1152/jappl.1975.39.1.174 1150585

[BIO053546C11] Cavanagh P. R. and Lafortune M. A. (1980). Ground reaction forces in distance running. *J. Biomech.* 13, 397-406. 10.1016/0021-9290(80)90033-0 7400169

[BIO053546C12] Chelly S. M. and Denis C. (2001). Leg power and hopping stiffness: relationship with sprint running performance. *Med. Sci. Sports Exerc.* 33, 326-333. 10.1097/00005768-200102000-00024 11224825

[BIO053546C13] Clark K. P. and Weyand P. G. (2014). Are running speeds maximized with simple-spring stance mechanics? *J. Appl. Physiol.* 117, 604-615. 10.1152/japplphysiol.00174.2014 25080925

[BIO053546C14] Clark K. P., Ryan L. J. and Weyand P. G. (2014). Foot speed, foot-strike and footwear: linking gait mechanics and running ground reaction forces. *J. Exp. Biol.* 217, 2037-2040. 10.1242/jeb.099523 24737756

[BIO053546C15] Clark K. P., Ryan L. J. and Weyand P. G. (2017). A general relationship links gait mechanics and running ground reaction forces. *J. Exp. Biol.* 220, 247-258. 10.1242/jeb.138057 27811299

[BIO053546C16] Clark K. P., Rieger R. H., Bruno R. F. and Stearne D. J. (2019). The National Football League combine 40-yd dash: how important is maximum velocity? *J. Strength Cond. Res.* 33, 1542-1550. 10.1519/JSC.0000000000002081 28658072

[BIO053546C17] Copaver K., Hertogh C. and Hue O. (2012). The effects of psoas major and lumbar lordosis on hip flexion and sprint performance. *Res. Q. Exerc. Sport* 83, 160-167. 10.1080/02701367.2012.10599846 22808701

[BIO053546C18] Deane R. S., Chow J. W., Tillman M. D. and Fournier K. A. (2005). Effects of hip flexor training on sprint, shuttle run, and vertical jump performance. *J. Strength Cond. Res.* 19, 615-621. 10.1519/00124278-200508000-00022 16095411

[BIO053546C19] Dorn T. W., Schache A. G. and Pandy M. G. (2012). Muscular strategy shift in human running: dependence of running speed on hip and ankle muscle performance. *J. Exp. Biol.* 215, 1944-1956. 10.1242/jeb.064527 22573774

[BIO053546C20] Farley C. T., Glasheen J. and McMahon T. A. (1993). Running springs: speed and animal size. *J. Exp. Biol.* 185, 71-86. 8294853 10.1242/jeb.185.1.71

[BIO053546C21] Gatesy S. M. and Biewener A. A. (1991). Bipedal locomotion: effects of speed, size and limb posture in birds and humans. *J. Zool.* 224, 127-147. 10.1111/j.1469-7998.1991.tb04794.x

[BIO053546C22] He J. P., Kram R. and McMahon T. A. (1991). Mechanics of running under simulated low gravity. *J. Appl. Physiol.* 71, 863-870. 10.1152/jappl.1991.71.3.863 1757322

[BIO053546C23] Kivi D. M., Maraj B. K. and Gervais P. (2002). A kinematic analysis of high-speed treadmill sprinting over a range of velocities. *Med. Sci. Sports Exerc.* 34, 662-666. 10.1249/00005768-200204000-00016 11932576

[BIO053546C24] Krzysztof M. and Mero A. (2013). A kinematics analysis of three best 100 m performances ever. *J. Hum. Kinet.* 36, 149-160. 10.2478/hukin-2013-0015 23717364 PMC3661886

[BIO053546C25] Kunz H. and Kaufmann D. A. (1981). Biomechanical analysis of sprinting: decathletes versus champions. *Br. J. Sports Med.* 15, 177-181. 10.1136/bjsm.15.3.177 7272662 PMC1858761

[BIO053546C26] Kuitunen S., Komi P. V. and Kyröläinen H. (2002). Knee and ankle joint stiffness in sprint running. *Med. Sci. Sports Exerc.* 34, 166-173. 10.1097/00005768-200201000-00025 11782663

[BIO053546C27] Macadam P., Cronin J. B. and Simperingham K. D. (2017). The effects of wearable resistance training on metabolic, kinematic and kinetic variables during walking, running, sprint running and jumping: a systematic review. *Sports Med.* 47, 887-906. 10.1007/s40279-016-0622-x 27638041

[BIO053546C28] Mann R. and Herman J. (1985). Kinematic analysis of Olympic sprint performance: men's 200 meters. *J. Appl. Biomech.* 1, 151-162. 10.1123/ijsb.1.2.151

[BIO053546C29] Mann R. and Murphy A. (2018). *The Mechanics of Sprinting and Hurdling*. Las Vegas, NV: CreateSpace Independent Publishing Platform.

[BIO053546C30] McGeer T. (1990). Passive bipedal running. *Proc. R. Soc. Lond. B Biol. Sci.* 240, 107-134. 10.1098/rspb.1990.0030 1972987

[BIO053546C31] McGowan C. P., Grabowski A. M., McDermott W. J., Herr H. M. and Kram R. (2012). Leg stiffness of sprinters using running-specific prostheses. *J. R. Soc. Interface* 9, 1975-1982. 10.1098/rsif.2011.0877 22337629 PMC3385759

[BIO053546C32] McMahon T. A. and Cheng G. C. (1990). The mechanics of running: how does stiffness couple with speed? *J. Biomech.* 23, 65-78. 10.1016/0021-9290(90)90042-2 2081746

[BIO053546C33] Mendiguchia J., Edouard P., Samozino P., Brughelli M., Cross M., Ross A., Gill N. and Morin J. B. (2016). Field monitoring of sprinting power–force–velocity profile before, during and after hamstring injury: two case reports. *J. Sports Sci.* 34, 535-541. 10.1080/02640414.2015.1122207 26648237

[BIO053546C34] Miyashiro K., Nagahara R., Yamamoto K. and Nishijima T. (2019). Kinematics of maximal speed sprinting with different running speed, leg length and step characteristics. *Front. Sports Active Living* 1, 37 10.3389/fspor.2019.00037 PMC7739839 33344960

[BIO053546C35] Munro C. F., Miller D. I. and Fuglevand A. J. (1987). Ground reaction forces in running: a reexamination. *J. Biomech.* 20, 147-155. 10.1016/0021-9290(87)90306-X 3571295

[BIO053546C36] Nagahara R., Matsubayashi T., Matsuo A. and Zushi K. (2014). Kinematics of transition during human accelerated sprinting. *Biol. Open* 3, 689-699. 10.1242/bio.20148284 24996923 PMC4133722

[BIO053546C37] Nagahara R., Kameda M., Neville J. and Morin J.-B. (2020). Inertial measurement unit- based hip flexion test as an indicator of sprint performance. *J. Sports Sci.* 38, 53-61. 10.1080/02640414.2019.1680081 31623521

[BIO053546C38] Nigg B. M., Bahlsen H. A., Luethi S. M. and Stokes S. (1987). The influence of running velocity and midsole hardness on external impact forces in heel-toe running. *J. Biomech.* 20, 951-959. 10.1016/0021-9290(87)90324-1 3693376

[BIO053546C39] Nilsson J. and Thorstensson A. (1989). Ground reaction forces at different speeds of human walking and running. *Acta Physiol. Scand.* 136, 217-227. 10.1111/j.1748-1716.1989.tb08655.x 2782094

[BIO053546C40] Novacheck T. F. (1998). The biomechanics of running. *Gait Posture* 7, 77-95. 10.1016/S0966-6362(97)00038-6 10200378

[BIO053546C41] Nummela A., Keränen T. and Mikkelsson L. O. (2007). Factors related to top running speed and economy. *Int. J. Sports Med.* 28, 655-661. 10.1055/s-2007-964896 17549657

[BIO053546C42] Rabita G., Dorel S., Slawinski J., Sàez-de-Villarreal E., Couturier A., Samozino P. and Morin J.-B. (2015). Sprint mechanics in world–class athletes: a new insight into the limits of human locomotion. *Scand. J. Med. Sci. Sports* 25, 583-594. 10.1111/sms.12389 25640466

[BIO053546C43] Raibert M. H. (1986). Symmetry in running. *Science* 231, 1292-1294. 10.1126/science.3945823 3945823

[BIO053546C44] Rubenson J., Lloyd D. G., Besier T. F., Heliams D. B. and Fournier P. A. (2007). Running in ostriches (Struthio camelus): three-dimensional joint axes alignment and joint kinematics. *J. Exp. Biol.* 210, 2548-2562. 10.1242/jeb.02792 17601959

[BIO053546C45] Samozino P., Rabita G., Dorel S., Slawinski J., Peyrot N., Saez de Villarreal E. and Morin J.-B. (2016). A simple method for measuring power, force, velocity properties, and mechanical effectiveness in sprint running. *Scand. J. Med. Sci. Sports* 26, 648-658. 10.1111/sms.12490 25996964

[BIO053546C46] Schache A. G., Blanch P. D., Dorn T. W., Brown N. A., Rosemond D. and Pandy M. G. (2011). Effect of running speed on lower limb joint kinetics. *Med. Sci. Sports Exerc.* 43, 1260-1271. 10.1249/MSS.0b013e3182084929 21131859

[BIO053546C47] Seyfarth A., Geyer H. and Herr H. (2003). Swing-leg retraction: a simple control model for stable running. *J. Exp. Biol.* 206, 2547-2555. 10.1242/jeb.00463 12819262

[BIO053546C48] Sides D. L. (2015). Kinematics and kinetics of maximal velocity sprinting and specificity of training in elite athletes. *PhD thesis*, University of Salford, UK.

[BIO053546C49] Thompson C. M. and Raibert M. H. (1989). Passive dynamic running. In *Dynamically Stable Legged Locomotion* (ed. Raibert M. H.), pp. 135-146. Cambridge, MA: MIT Artificial Intelligence Laboratory.

[BIO053546C50] Udofa A. B., Clark K. P., Ryan L. J. and Weyand P. G. (2019). Running ground reaction forces across footwear conditions are predicted from the motion of two body mass components. *J. Appl. Physiol.* 126, 1315-1325. 10.1152/japplphysiol.00925.2018 30763160

[BIO053546C51] Weyand P. G., Sternlight D. B., Bellizzi M. J. and Wright S. (2000). Faster top running speeds are achieved with greater ground forces not more rapid leg movements *J. Appl. Physiol.* 89, 1991-1999. 10.1152/jappl.2000.89.5.1991 11053354

[BIO053546C52] Weyand P. G., Sandell R. F., Prime D. N. L. and Bundle M. W. (2010). The biological limits to running speed are imposed from the ground up. *J. Appl. Physiol.* 108, 950-961. 10.1152/japplphysiol.00947.2009 20093666

[BIO053546C53] Winter D. A. (1990). *Biomechanics and Motor Control of Human Movement*, 2nd edn. New York: John Wiley & Sons, Inc..

