# Supplementary material for: ‘Whip from the hip’: thigh angular motion, ground contact mechanics, and running speed
Source: Biol Open. 2020 Oct 21;9(10):bio053546. doi: 10.1242/bio.053546 (PMC7595692; doi:10.1242/bio.053546)
Supplement: Supplementary information [file biolopen-9-053546-s1.pdf]

SUPPLEMENTARY INFORMATION

Table S1. Subject descriptive characteristics, categorized by sex and athletic background.

Group Description	Age (years)	Height (m)	Leg Length (m)	Mass (kg)	BMI (kg m^{-2})
**All Males (*n* = 20)**	**21.6 ± 0.5**	**1.80 ± 0.01**	**0.94 ± 0.01**	**79.7 ± 3.0**	**24.6 ± 0.7**
Male Track & Field Athletes (*n* = 9)	21.6 ± 0.8	1.78 ± 0.01	0.93 ± 0.01	73.4 ± 2.0	23.1 ± 0.5
Male Team Sport Athletes (*n* = 5)	20.2 ± 0.4	1.83 ± 0.03	0.97 ± 0.03	87.0 ± 8.6	25.9 ± 1.7
Male Recreationally Trained (*n* = 6)	22.7 ± 1.2	1.79 ± 0.03	0.92 ± 0.02	82.9 ± 5.7	25.7 ± 1.4
**All Females (*n* = 20)**	**21.7 ± 0.4**	**1.67 ± 0.02**	**0.89 ± 0.01**	**59.0 ± 1.4**	**21.1 ± 0.5**
Female Track & Field Athletes (*n* = 6)	23.0 ± 1.0	1.72 ± 0.02	0.91 ± 0.01	60.6 ± 2.0	20.4 ± 0.4
Female Team Sport Athletes (*n* = 8)	21.0 ± 0.3	1.62 ± 0.03	0.88 ± 0.02	58.4 ± 2.9	22.2 ± 0.6
Female Recreationally Trained (*n* = 6)	21.3 ± 0.3	1.70 ± 0.03	0.90 ± 0.02	58.2 ± 2.2	20.2 ± 1.1

All values are group means ± standard error of the mean (s.e.m.).

Table S2. Additional thigh angular kinematic variables across speeds, with trials categorized by percentage top speed.

	Slow<br><75% Top Speed<br>(*n* = 44 trials)	Intermediate<br>75-93% Top Speed<br>(*n* = 48 trials)	Fast<br>>93% Top Speed<br>(*n* = 62 trials)
θ_{td} (rad, *deg*)	0.49 ± 0.01<br>*27.9 ± 0.6*	0.55 ± 0.01†<br>*31.7 ± 0.7*	0.61 ± 0.01*†<br>*35.0 ± 0.6*
θ_{to} (rad, *deg*)	0.36 ± 0.02<br>*20.7 ± 1.0*	0.43 ± 0.02†<br>*24.5 ± 1.0*	0.44 ± 0.01†<br>*25.3 ± 0.7*
θ_{ext} (rad, *deg*)	0.41 ± 0.02<br>*23.8 ± 1.2*	0.51 ± 0.02†<br>*29.2 ± 1.0*	0.55 ± 0.01†<br>*31.7 ± 0.8*
θ_{flex} (rad, *deg*)	0.78 ± 0.03<br>*44.9 ± 1.9*	0.98 ± 0.03†<br>*56.2 ± 1.6*	1.09 ± 0.03*†<br>*62.5 ± 1.5*

All angular variables are absolute values. Values are means ± standard error of the mean (s.e.m.).

Thigh angular variables: thigh angular position at touchdown (θ_{td}), thigh angular position at takeoff (θ_{to}), peak thigh extension during flight (θ_{ext}), and peak thigh flexion during flight (θ_{flex}).

† indicates significantly different than Slow speed ($p < 0.05$)
* indicates significantly different than Intermediate speed ($p < 0.05$)

Biology Open • Supplementary information

Table S3. Additional thigh angular kinematic variables across top speed trials, with subjects categorized based on sex and top speed.

	Fast Males (n = 10)	Slow Males (n = 10)		Fast Females (n = 10)	Slow Females (n = 10)
θ_{td} (rad, *deg*)	0.62 ± 0.02<br>*35.4 ± 1.2*	0.62 ± 0.02<br>*35.4 ± 1.3*		0.59 ± 0.04<br>*34.0 ± 2.0*	0.59 ± 0.01<br>*34.0 ± 0.7*
θ_{to} (rad, *deg*)	0.40 ± 0.03<br>*23.2 ± 1.6*	0.47 ± 0.03<br>*27.2 ± 1.6*		0.47 ± 0.03<br>*26.7 ± 1.7*	0.50 ± 0.02<br>*28.7 ± 1.4*
θ_{ext} (rad, *deg*)	0.50 ± 0.03<br>*28.6 ± 1.9*	0.59 ± 0.04<br>*33.8 ± 2.5*		0.59 ± 0.03<br>*33.7 ± 1.7*	0.62 ± 0.03<br>*35.5 ± 1.9*
θ_{flex} (rad, *deg*)	1.23 ± 0.07<br>*70.3 ± 3.8*	1.04 ± 0.07<br>*59.6 ± 3.8*		1.06 ± 0.06<br>*60.6 ± 3.6*	0.93 ± 0.01<br>*53.2 ± 0.7*

All angular variables are absolute values. Values are means ± standard error of the mean (s.e.m.).

Thigh angular variables: thigh angular position at touchdown (θ_{td}), thigh angular position at takeoff (θ_{to}), peak thigh extension during flight (θ_{ext}), and peak thigh flexion during flight (θ_{flex}).

† indicates significantly different ($p < 0.05$), Faster Males vs. Slower Males
* indicates significantly different ($p < 0.05$), Faster Females vs. Slower Females

Biology Open • Supplementary information

Fig. S1. Evaluation of the motion capture method for determining running speed. Running speed in the 31 to 39 m field of view (*n* = 154) measured by the radar gun is compared to speed determined by the motion capture method, with dashed Line of Identify (x = y).

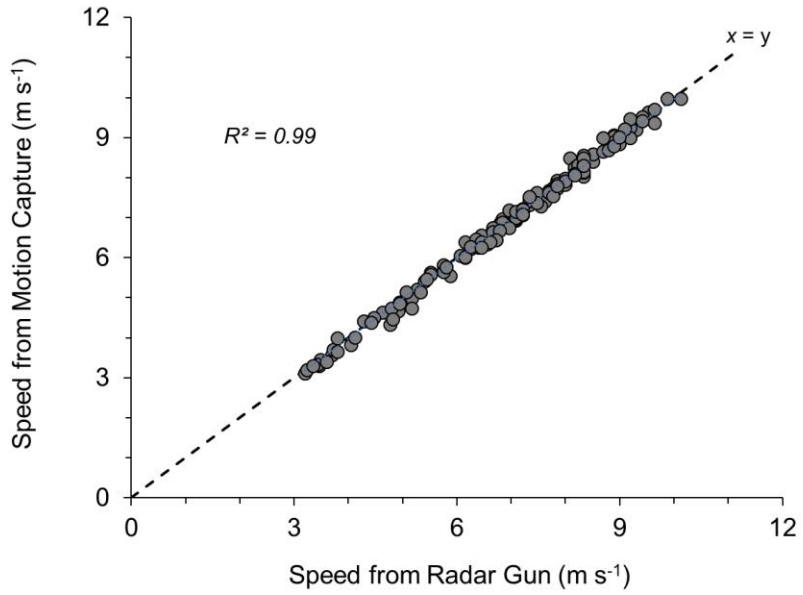

Biology Open • Supplementary information

Fig. S2. Evaluation of the motion capture method for determining ground contact time (T_c). For the sub-set of trials (n = 95) used to complete this validation, T_c measured by the force plate is plotted compared to T_c determined by the motion capture method, with dashed Line of Identify (x = y).

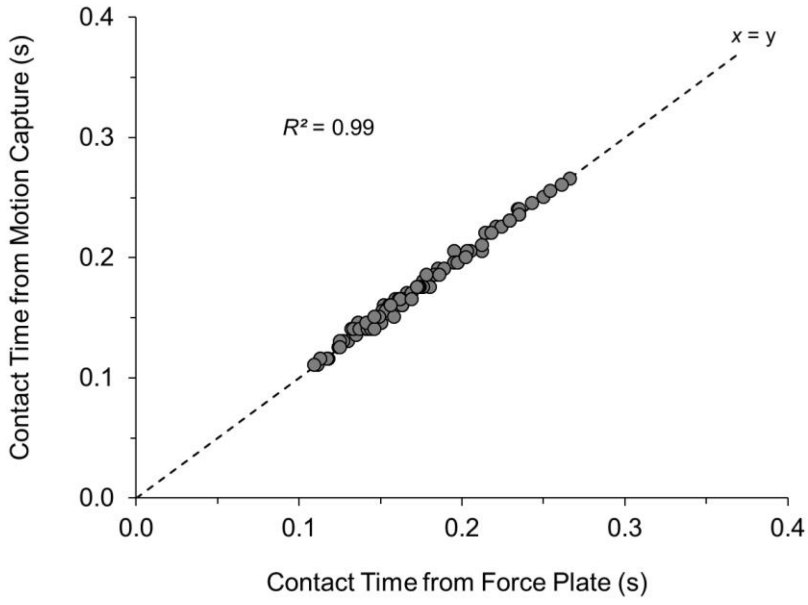

Biology Open • Supplementary information
